# Identifying *cis*- and *trans*-acting single-nucleotide polymorphisms controlling lymphocyte gene expression in humans

**DOI:** 10.1186/1753-6561-1-s1-s7

**Published:** 2007-12-18

**Authors:** Pingzhao Hu, Hui Lan, Wei Xu, Joseph Beyene, Celia MT Greenwood

**Affiliations:** 1 Program in Genetics and Genome Biology, The Hospital for Sick Children Research Institute, 15-706 TMDT, 101 College Street, Toronto, Ontario, M5G 1L7, Canada; 2 Department of Computer Science, University of Toronto, 10 Kings College Road, Toronto, Ontario, M5S 3G4, Canada; 3 Department of Public Health Sciences, University of Toronto, 155 College Street, Toronto, Ontario, M5T 3M7, Canada; 4 Department of Biostatistics, Princess Margaret Hospital, Toronto, Ontario, M5G 2M9, Canada; 5 Program in Child Health Evaluative Sciences, The Hospital for Sick Children Research Institute, 555 University Ave, Toronto, Ontario, M5G 1X8, Canada

## Abstract

Assuming multiple loci play a role in regulating the expression level of a single phenotype, we propose a new approach to identify *cis*- and *trans*-acting loci that regulate gene expression. Using the Problem 1 data set made available for Genetic Analysis Workshop 15 (GAW15), we identified many expression phenotypes that have significant evidence of association and linkage to one or more chromosomal regions. In particular, six of ten phenotypes that we found to be regulated by *cis*- and *trans*-acting loci were also mapped by a previous analysis of these data in which a total of 27 phenotypes were identified with expression levels regulated by *cis*-acting determinants. However, in general, the *p*-values associated with these regulators identified in our study were larger than in their studies, since we had also identified other factors regulating expression. In fact, we found that most of the gene expression phenotypes are influenced by at least one *trans*-acting locus. Our study also shows that much of the observable heritability in the phenotypes could be explained by simple single-nucleotide polymorphism associations; residual heritability was reduced and the remaining heritability may represent complex regulation systems with interactions or noise.

## Background

**Figure 1:**
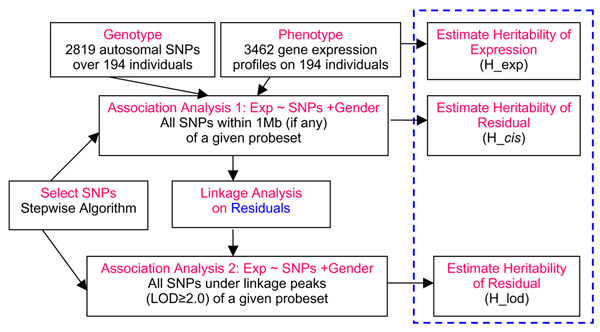
Flow chart showing the analytical strategies we used to identify *cis*- and *trans*-acting regulators.

Gene expression levels of many genes show natural variation in humans [1,2]. In an individual, the expression levels of a highly variable gene can be treated as a 'phenotype', possibly influenced by genetic determinants. Recent studies have shown that expression levels may be influenced by single-nucleotide polymorphism (SNP) alleles [1-3]. These mapping efforts have identified quantitative trait loci (QTLs) that may be in the gene's own regulatory regions (*cis*-acting QTLs) as well as elsewhere in the genome (*trans*-acting QTLs) using both linkage [1] and association analysis [2,3]. For the association analysis, Stranger et al. [3] examined all possible combinations of gene expression phenotype/marker genotype combinations, whereas Cheung et al. [2] examined only gene expression phenotype/genotype combinations under linkage peaks identified in the study by Morley et al. [1]. Here we take the advantages of these analysis methods and propose a new analysis strategy for the 3462 gene expression measurements on members of 14 CEPH Utah families, provided to participants in Genetic Analysis Workshop 15 (GAW15), as shown in Figure 1. The main differences between our method and previous ones are: 1) we assumed that there may be multiple loci regulating the expression levels of a single gene and used stepwise regression analysis to look for additive effects of the SNPs; 2) we examined the evidence for linkage to residuals of linear regression analysis adjusting for gender and *cis*-SNPs, rather than to raw expression intensities, and 3) we evaluated the changing patterns of expression heritability and residual heritability.

## Methods

### Phenotypes and genotypes

Expression levels for 3554 genes, taken from the lymphoblastoid cell lines of 194 members of 14 CEPH Utah families, were made available for GAW15 [1]. Of these expression measures, 92 were missing either chromosome number or start or end of chromosomal location information in the Affymetrix annotation table , so we focused on the remaining 3462 gene expression traits. The genotypes for 2819 autosomal SNPs for the same individuals were generated by The SNP Consortium .

### Definition of *cis*- and *trans*-acting regulators

*cis*-Regulatory variants were defined as SNPs either within a gene, up to 1 Mb proximal to the start of the gene, or up to 1 Mb distal to the end of the gene. *trans*-Regulatory polymorphisms are defined as all SNPs elsewhere in the genome. Physical locations of probe sets were obtained from the Affymetrix annotation table . The Rutgers map was used to establish a correspondence between the megabase locations on the physical map and the genetic map . Markers that could not be mapped using Rutgers map, but that were located between physically anchored markers, were placed on the genetic map by linear interpolation.

### Identification of *cis*- and *trans*-acting regulators

Three steps were used to identify *cis*- and *trans*-regulatory polymorphisms (See Figure 1). 1) For each probeset, we first identified SNPs in or close to (1 Mb) the probe set (*cis*-SNPs), and then we fit a linear regression model containing gender and the *cis*-SNPs as covariates. If no *cis*-SNPs were identified for the probe set, we used only gender as a covariate; if more than one *cis*-SNP was identified, a stepwise algorithm based on the Akaike information criterion (AIC) was applied to choose a predictive set of *cis*-SNPs; if no SNPs were kept after running the stepwise algorithm, we forced in the SNP with the smallest *p*-value. The SNPs were coded 0, 1, and 2, representing homozygous rare, heterozygous, and homozygous common genotypes, respectively. Within-family dependence was not modelled, although we did some sensitivity analyses examining the effect of this assumption (see Discussion). We report the nominal, parametric *p*-values for the test of no association for each SNP (β = 0). 2) Residuals were obtained from the previous linear models containing gender and *cis*-SNPs (if any). Genome-wide multipoint linkage analysis was then performed on the residuals using the MERLIN-REGRESS command in the statistical genetics software MERLIN [4]. 3) For each of the gene expression phenotypes, we fit a new linear model to identify SNPs under the linkage peaks of step (2) (logarithm of the odds (LOD) ≥ 2.0) that influence gene expression, and also included gender. These were primarily *trans*-SNPs because the linkage models used residuals that had already been adjusted for *cis*-SNPs. We evaluated which of these SNPs significantly and independently predicted gene expression phenotype by using the stepwise procedure with AIC to choose the optimal set of SNPs in the model.

### Heritability estimation

The variance components analysis in MERLIN was used to estimate heritability based on: 1) raw gene expression profiles (H_exp); 2) residuals to a stepwise regression analysis containing *cis*-SNPs (if any) and gender (H_cis); 3) residuals to a stepwise regression analysis containing SNPs (if any) that have LOD of at least 2.0 and gender (H_lod).

## Results

We identified 2176 out of 3462 expression phenotypes where there was at least one *cis*-SNP. Table 1 (Step 1) shows the number of expression phenotypes that had *cis*-SNPs nearby, and the number that were associated with the phenotypes. There were 1286 expression phenotypes without an associated *cis*-SNP. Of the 2176 phenotypes with *cis*-SNPs, the stepwise model chose only one SNP as associated for 78% (1697 out of 2176) of the phenotypes, although 81% (1763 out of 2176) of the phenotypes had more than one nearby SNP. Using a definition of significance of *p *= 0.01, 288 phenotypes were associated with at least one significant *cis*-regulator, and of these, 43 phenotypes were associated with two significant *cis*-regulators. If a more stringent significant level, say *p *= 0.001, is used, the number of significant *cis*-SNP drops dramatically (Table 1).

**Table 1:** Distribution of the number of expression phenotypes with different number of SNPs in the regression models of steps 1 and 3 of our three-step method

Step	No. SNPs	0	1	2	3	4	5	6	7	8	9	≥10
1	Available^a^	1286	413	310	614	417	148	169	59	16	16	14
	AIC^b^	1286	1697	350	107	17	3	1	1	0	0	0
	*p *≤ 0.01^c^		245	43	0	0	0	0	0	0	0	0
	*p *≤ 0.001^c^		88	5	0	0	0	0	0	0	0	0
												
3	Linkages^d^	294	81	76	118	141	102	101	102	110	91	2264
	AIC	294	664	362	295	270	236	187	147	127	101	645
	*p *≤ 0.01^c^		930	433	188	101	58	38	21	11	11	32
	*p *≤ 0.001^c^		572	151	60	25	12	10	3	6	2	2

^a^"Available" denotes the number of phenotypes with the given number of available *cis*-SNPs in Step 1.

^b^"AIC" denotes the number of phenotypes where the given number of SNPs was retained by the stepwise linear regression model.

^c^The number of phenotypes among those in the previous line in which the *p*-values of SNP associated with the phenotypes are less than or equal to the given significance level.

^d^"Linkages" denotes the number of phenotypes linked (LOD > 2.0) to the given number of SNPs.

We then performed linkage analysis using residuals for the 3462 expression phenotypes derived from the fitted association models using the associated *cis*-SNPs and gender. Morley et al. [1] defined two levels of significance: *p *= 3.7 × 10^-5 ^(LOD ~ 3.4), and *p *= 4.3 × 10^-7 ^(LOD ~ 5.3). Using the same thresholds, we identified 1556 and 337 expression phenotypes with at least one marker showing evidence for linkage beyond these thresholds, respectively. In comparison, Morley et al. [1] identified 984 and 142 phenotypes, respectively, with at least one region of linkage at these two levels. We found many expression phenotypes whose regulation mapped to shared hotspots on chromosomes 9, 11, 13, 14, and 20.

We then performed a second set of stepwise linear regression analyses for the expression phenotypes, including gender and SNPs that showed LOD scores ≥ 2.0. There were 3034 of these 3462 phenotypes with at least one linkage peak. Given a *p*-value threshold of 0.01, we found 930 of the 3034 phenotypes were significantly associated with one marker and another 893 phenotypes were significantly associated with more than one marker. The remaining 1639 phenotypes showed no significant association with any marker. Again, if a more stringent significance level is used, the number of identified significant *trans*-SNPs will be decreased, but not by as much as in Step 1. Focusing on the most significant SNP for each phenotype, we found that 1514 (83.0%) expression phenotypes are most strongly influenced by a *trans*-acting transcriptional regulator.

Of the 337 phenotypes with at least one LOD score over Morley's second threshold (LOD ≥ 5.3), 10 (3.0%) were found to have a *cis*-acting as well as at least 1 *trans*-acting regulator (Table 2), and 6 of these (VAMP8, GSTM1, GSTM2, IRF5, DDX17, and CHI3L2) were also identified by Morley et al. [1] (see also Cheung et al. [2], Table 1). It is also interesting to find 3 (PARP4, GSTM1 and IRF5) of the 10 phenotypes show copy number variation in healthy individuals [5], suggesting there is a potential relationship between gene regulation and copy number variation. Of the remainder, 95 (28.2%) are influenced by only one *trans*-regulator, and other 232 (68.8%) of the phenotypes show associations with more than one *trans*-regulator. As can be seen from Table 2, among the 10 phenotypes having strong linkage evidence to both *cis*-regulators and *trans*-regulators, some of the most strongly linked *cis*-SNPs also show evidence of association in Step 1. For these 10 phenotypes, we also observed that the variation explained in the second association analysis (Step 3) is greatly increased compared with that in the first association analysis (Step 1). According to Step 3, between 2 and 8 polymorphisms can the majority of the phenotypic variance; for gene *VAMP8 *(Table 2), three SNPs explain 99.9% of the variance.

**Table 2:** Ten phenotypes whose expression level is significantly regulated by both *cis*- and *trans*-acting determinants

		*Cis*-Association analysis (Step 1)		Linkage analysis of residuals (Step 2) – Signals at *cis*-SNPs		Association analysis under linkage peaks (Step 3)	
				
Gene Symbol	Location	*p*-value for *cis-*SNP with peak LOD score	Variation explained (*R*^2^%)	cis-SNP with peak LOD score	Peak (*cis*) LOD score	(R^2^%)	No. of SNPs in model
PARP4^a^	13q11	0.023	3.3	rs735770	6.21	80.2	4
VAMP8^b^	2p12-p11.2	0.003	1.2	rs1432265	7.8	99.9	3
ITGB1BP1	2p25.2	0.051	2.4	rs1003653	10.56	55.3	5
TPP2	13q32-q33	0.693	0.2	rs1412953	6.01	99.3	3
GSTM2^b^	1p13.3	0.001	8.9	rs559479	9.22	86.8	4
GSTM1^a, b^	1p13.3	0.024	7.1	rs15864	6.53	32.3	8
IRF5^a, b^	7q32	0.606	0.2	rs754386	6.1	98.9	5
DDX17^b^	22q13.1	0.01	19.3	rs2064088	10.89	70.8	6
CHI3L2^b^	1p13.3	0.086	10.1	rs1264898	9.48	64.9	2
PEX6	6p21.1	0.004	11.6	rs1537638	7.2	66.7	5

This table shows the result from the *cis*-SNP association analysis (step 1) that corresponds to the largest *cis*-LOD score in step 2, as well as the total variance explained from step 3, for each of these expression measures.

^a^Regions of these genes show copy number variation .

^b^These genes were also identified by Morley et al. [1] and are shown in Table 1 of Cheung et al. [2].

Table 3 shows the distribution of heritability for the 3462 phenotypes which were analyzed in three ways (see Figure 1). It can be seen that residual heritability decreases from H_exp to H_cis to H_lod, showing that the gene expression patterns can be partially explained by one or more SNPs. Nine percent of the raw phenotypes had heritability over 0.4, whereas only 5.5% of the H_lod residuals had heritability over 0.4.

**Table 3:** Distribution of heritability estimates for expression phenotypes

Heritability interval	H_exp	H_*cis*	H_lod
0.00–0.20	1758	1937	2116
0.21–0.40	1392	1252	1154
0.41–0.60	276	238	165
0.61–0.80	33	29	25
0.81–1.00	3	6	2

## Discussion & conclusion

Genetic and environmental factors influence gene expression through complex pathways. Therefore, useful insight can be gained by considering jointly the effects of covariates and several SNPs when examining factors influencing gene expression. We included gender in all models and it was highly significant in many models (data not shown). It would also be interesting to include age to examine more complex genetic relationships. We also performed linkage analysis on residuals to models containing gender and *cis*-SNP effects, rather than performing linkage analysis on raw expression intensities. This approach may reduce residual variance and hence make it possible to identify additional factors influencing expression.

We allowed multiple SNPs to be considered for each linear regression and hence we identified phenotypes that are associated with several different SNPs in different parts of the genome. For some genes, a very large proportion of the variability was explained by a combination of several SNPs (see Table 2). Often, there may be several nearby SNPs that all show univariate associations with an expression phenotype. Correlations between these SNPs mean that, often, only some of these SNPs would be retained by the stepwise regression – a more parsimonious model can capture the association in a genomic region. We identified some of the same *cis*-controlled phenotypes as Morley et al. [1] and Cheung et al. [2], but our statistical significance was reduced relative to theirs. This may be a consequence of including several SNPs as well as gender in each model, in conjunction with a small sample size. We did not examine interactions between SNPs or genes; however, it would be interesting to model interactions between *cis*-SNPs in the regression analysis to explore joint effects. Despite performing linkage on residuals, we sometimes found linkage to regions near the *cis*-SNPs, probably due to multi-marker linkage patterns, incompletely explained by allelic variability.

Although we used simple linear regression to explore SNP associations and did not correct for additional familial dependence in this analysis, we compared our first stage results with generalized estimating equation (GEE) models using only the 413 phenotypes where there was exactly one *cis*-SNP available. Our regression analysis identified 15 of these phenotypes to have a significant *cis*-SNP (*p *≤ 0.01) while the GEE model identified only 10 with one significant *cis*-SNP (*p *≤ 0.01). Five of these phenotypes were identified by both methods. By ignoring familial clustering, we may have *p*-values that are too small. It would be worth fitting random effect models or GEE models to all the data, as well as models that are robust to non-normal distributions. However, the number of families here is quite small and any general conclusions would be better drawn from a larger sample.

Conceptually, we showed that a sizeable proportion of the observable heritability could be explained by simple SNP associations for these lymphoblastoid expression phenotypes. The 5.5% of the phenotypes where residual heritability remained over 0.4 may be influenced by complex regulation systems.

## Competing interests

The author(s) declare that they have no competing interests.

## Authors' contributions

CMTG proposed the methods. PH and HL analyzed the data. PH drafted the manuscript. WX and JB participated in the data analysis. JB directed GEE analysis. All authors revised the manuscript and read and approved the final manuscript.
